# Barriers to accessing health services during the COVID-19 pandemic in Poland: A nationwide cross-sectional survey among 109,928 adults in Poland

**DOI:** 10.3389/fpubh.2022.986996

**Published:** 2022-09-08

**Authors:** Paulina Mularczyk-Tomczewska, Adam Zarnowski, Mariusz Gujski, Mateusz Jankowski, Iwona Bojar, Artur Wdowiak, Jan Krakowiak

**Affiliations:** ^1^Department of Public Health, Medical University of Warsaw, Warsaw, Poland; ^2^School of Public Health, Center of Postgraduate Medical Education, Warsaw, Poland; ^3^Department of Women's Health, Institute of Rural Health in Lublin, Lublin, Poland; ^4^Chair of Obstetrics and Gynecology, Faculty of Health Sciences, Medical University of Lublin, Lublin, Poland; ^5^Department of Social Medicine, Medical University of Lodz, Lodz, Poland

**Keywords:** access to healthcare, medical care, barriers, COVID-19, pandemic, health services research, organization of health care

## Abstract

**Introduction:**

The COVID-19 pandemic has significant socio-economic implications for numerous industries, including healthcare. Disruptions of essential health services were reported by nearly all countries around the world. A detailed assessment of the healthcare uptake is necessary to estimate the potential health effects of the COVID-19 pandemic on the population. This study aimed to assess the prevalence of barriers to accessing health services during the COVID-19 pandemic in Poland as well as to identify factors associated with the disturbed access to healthcare during the pandemic.

**Methods:**

This cross-sectional survey was carried out among Internet users in Poland using the computer-assisted web interview technique. Data were collected between October and December 2021. The questionnaire included 32 questions on sociodemographic characteristics, the COVID-19 pandemic, health status, daily habits as well as the use of healthcare during the COVID-19 pandemic.

**Results:**

Data were obtained from 102928 adults, the mean age was 48.0 ± 14.2 years, and 57.2% were females. Most of the respondents had visited a doctor during the past 12 months (70.4%). Almost half of adults in Poland (49.7%) reported barriers to access health services in the past 12 months. Out of 51,105 respondents who had experienced barriers to accessing health services during the COVID-19 pandemic, only 54.3% had visited a doctor in the past 12 months. Long waiting time (39.5%) and temporary closure of healthcare facilities/transformation into a COVID-19 dedicated center (28.8%) were the most common barriers indicated by the respondents. In multivariable logistic regression, female gender, age 18–49 years, lack of higher education, living in cities between 51,000 and 200,000 residents or above 500,000 residents, and having at least one chronic disease were significantly (*p* < 0.05) associated with higher odds of experiencing barriers to accessing health services during the COVID-19 pandemic.

**Conclusions:**

This study confirmed that the COVID-19 pandemic has worsened access to health services in Poland. During the pandemic, new barriers to accessing health services, such as the temporary closure of healthcare facilities for non-COVID patients were revealed. Findings from this study provided patients' perspectives on barriers to accessing health services in Poland that may be used by policymakers to reduce health inequalities.

## Introduction

Coronavirus disease is an infectious disease caused by the SARS-CoV-2 virus (1, 2). On 11 March 2020, the World Health Organization (WHO) declared the COVID-19 outbreak a global pandemic (2). As of 30 June 2022, more than 552 million COVID-19 cases were reported globally (3). The COVID-19 pandemic posed a significant challenge for healthcare professionals as well as policymakers (4, 5). To mitigate the early spread of the COVID-19 pandemic, numerous countries had implemented anti-epidemic measures, such as lockdowns, distance learning at schools and universities, temporary suspension of the activities of public institutions, and as well re-organization of the method of managing healthcare resources (6, 7). Massive outbreaks of COVID-19 lead to a sudden flow of patients into emergency departments, and a rapid increase in the number of hospitalizations of patients that requires isolation and specialistic medical care (8, 9).

The COVID-19 pandemic has significant socio-economic implications for numerous industries, including healthcare (10). The implementation of teleconsultations in outpatient clinics (especially in primary care) sped up significantly (11). Moreover, the duration of the research and development process was shortened due to the urgent need for the implementation of COVID-19 prevention and treatment methods (12). Moreover, the COVID-19 pandemic forced the need to change the resource management in the healthcare system, especially in countries with a low number of healthcare workers.

Findings from the WHO report on health services during the COVID-19 pandemic showed, that disruptions of essential health services were reported by nearly all countries around the world (13). All types of health services were affected, including essential services for communicable diseases, non-communicable diseases, mental health, reproductive health, and child/adolescent health (13). According to the WHO estimates, emergency services were the least disrupted during the COVID-19 pandemic (13).

Poland is the fifth most populous European Union (EU) member state, with more than 38 million inhabitants. The first laboratory-confirmed COVID-19 case in Poland was reported on 4 March 2020 (14). As of 30 June 2022, Poland reported more than 6 million COVID-19 cases and 116 thousand COVID-related deaths (3). However, the dynamics of the COVID-19 pandemic differ across the regions within the country (15). The COVID-19 burden in Poland is one of the highest in the EU (3). Public government response to the COVID-19 pandemic has changed depending on the current guidelines of international health organizations and the current state of medical knowledge (16). Nevertheless, one of the goals of the anti-epidemic strategy in Poland was to ensure access to medical care for patients with COVID-19 (especially those with severe COVID-19).

To increase the capacity of healthcare in Poland, in 2020 selected general hospitals were transformed into single-purpose hospitals for infectious diseases (COVID-19 dedicated hospitals) (8, 16). Moreover, between 2020 and 2021, selected hospital wards (mostly internal medicine) were transferred into COVID-19 dedicated wards (8). What's more, in 2021, field hospitals have been prepared (mostly in sports facilities e.g., stadiums or exhibition halls) in each administrative region within the country. According to the Organization for Economic Co-operation and Development statistics, the number of medical doctors and nurses per 1,000 inhabitants in Poland is the lowest when compared to other European countries (17). Between April and July 2020, to reduce the risk of SARS-CoV-2 transmission in healthcare settings, healthcare workers were obligated to work in a single medical center (16). Moreover, in 2020, a markable proportion of general practitioners worked remotely and patients may visit the doctor only *via* teleconsultation (18). In 2020 and 2021, planned surgeries, non-emergency hospital admission as well as outpatient visits have been significantly reduced or canceled to reduce the risk of SARS-CoV-2 transmission between healthcare professionals, patients, and their families (18, 19). Hospital wards transformation into COVID-19 dedicated centers, temporary closure of healthcare facilities and transition into e-Health services as well as limited mobility due to lockdowns may have a significant impact on access to health services during the COVID-19 pandemic. However, the percentage of the population who expected the abovementioned barriers to access health services may vary between the countries and healthcare systems.

Healthcare in Poland is based on mandatory health insurance (20). This insurance is mandatory for all employed individuals (occupationally active status, regardless of the time and type of employment) (20). Moreover, the government is obliged to provide free health care services to young children, pregnant, students, disabled people, and the elderly (21). All insured individuals have access to a broad range of health services, including primary health services as well as specialized medical services like ambulatory care or hospital care (20, 21). In recent years, additional (private) health insurance, which complements the standard public (mandatory) insurance, has been gaining popularity (21). Supplementary private health insurance is often offered to employees as a non-wage benefit and promoted as providing faster access to specialist care (20, 21). In general, private medical facilities in Poland are perceived as better organized, with short waiting times, and more comprehensive access to health services when compared to public facilities (21).

A decrease in healthcare uptake during the COVID-19 pandemic may contribute to a health debt, and thus delay the diagnosis and treatment of diseases (22). Moreover, barriers to accessing health services during the pandemic may deepen health inequalities (23). However, there are limited data on health services during the COVID-19 pandemic in Poland, mostly focused on hospital admissions (23–25). Rajwa et al. (24) showed that between 2019 and 2020, the number of urological visits in Poland decreased by 22.4% and the number of urologic urgent admissions decreased by 11.8%. Grudziaz-Sekowska et al. (25) observed a significant decrease (−27% between 2020 and 2019) in the number of hospitalizations of children with type 1 diabetes. A detailed assessment of the healthcare uptake is necessary to estimate the potential health effects of the COVID-19 pandemic on the population.

Therefore, this study aimed to assess the prevalence of barriers to accessing health services during the COVID-19 pandemic in Poland as well as to identify factors associated with the disturbed access to healthcare during the pandemic.

## Materials and methods

### Study design and population

This cross-sectional survey was carried out among Internet users in Poland using the computer-assisted web interview (CAWI) technique. Data were collected between October and December 2021. The study questionnaire was distributed through websites operated by the Wirtualna Polska Media SA – one of the leading Polish-language media houses, with more than 13 million individual users every month (26). Information about the study (with reference link) was placed on advertising/information banners on the various websites (news pages, lifestyle, and health subpages). The study questionnaire was available on the dedicated project website “Think about yourself - we check the health of Poles in a pandemic” carried out by the Medical University of Warsaw, Wirtualna Polska Media SA, and the HomeDoctor Sp. z o.o. (26). All the fully completed questionnaires filled during the study period were included in the analysis (without any restrictions on the maximum sample size).

Participation in the study was voluntary and anonymous. Each participant was informed about the scope of the study as well as its course. Informed consent was collected from all the participants. This study was approved by the Ethical Review Board at the Medical University of Warsaw, Warsaw, Poland (no. AKBE/149/2021).

### Measures

A self-prepared questionnaire was used (27). The questionnaire was developed by experts in the field of public health, epidemiology, and general medicine. The Scientific Board of the project entitled “Think about yourself - we check the health of Poles in a pandemic” (26) was asked to analyze and indicate the potential impact of the pandemic and lockdowns on the use of health services in Poland. Moreover, a literature review on the impact of the COVID-19 pandemic on the organization of the healthcare systems and health services was carried out to identify the potential barriers to accessing health services that may be addressed in the study questionnaire (5, 8–11). Findings from the expert's consensus and the literature review were analyzed by the authors and used to develop a final version of the study questionnaire. The primary version of the questionnaire was used in a pilot study among 10 adults in Poland (aged 18–65 years). These individuals filled out the questionnaire twice, 5 days apart. The responses provided in each wave were compared. After the pilot study, 3 questions were rewritten to clarify their meaning.

The questionnaire included 32 questions on sociodemographic characteristics, the COVID-19 pandemic, health status, lifestyle, daily habits, preventive screening as well as the use of healthcare during the COVID-19 pandemic.

The use of healthcare: Respondents were asked about the use of healthcare during the COVID-19 pandemic, using the question: “In the last 12 months, have you visited a doctor or healthcare facility because of your or your child's illness or health condition? (yes/no).”

Barriers to accessing health services during the COVID-19 pandemic: All the respondents were asked about the potential barriers to accessing health services during the COVID-19 pandemic, using the question: “In the last 12 months, have you had to see a doctor, but this was not possible for any of the following reasons: (1) the healthcare facility was closed due to the COVID-19 pandemic or transferred into a COVID-19 dedicated center; (2) too long waiting time; (3) financial barrier; (4) distance to the medical facility or problems with transport to the doctor (yes/no).”

Respondents who indicated at least one barrier were classified as the group that had experienced barriers to accessing health services during the COVID-19 pandemic.

### Data analysis

The data were analyzed with SPSS version 28 (IBM Corp, Armonk, USA). Frequencies and proportions showed the distribution of categorical variables. Cross-tabulations and chi-squared tests were used to compare categorical variables.

Associations between personal characteristics (age, gender, educational level, place of residence, occupational status (currently employed or self-employed were defined as active), health status (presence of at least one chronic disease), private health insurance coverage, and accessing health services during the COVID-19 pandemic (visiting a doctor in the past 12 months and experiencing barriers to accessing health services) were analyzed using multivariable logistic regression models. The strength of association was measured by the odds ratio (OR) and 95% confidence intervals (CIs). The level of statistical significance was set at *p* < 0.05.

## Results

### Characteristics of the study population

Data were obtained from 102,928 adults, mean age 48.0 ± 14.2 years, 57.2% females (27). Among the respondents, 42.4% had chronic diseases and 41.1% had private health insurance. Most of the respondents had visited a doctor during the past 12 months (70.4%). Females, older respondents, those with higher education, currently unemployed individuals (passive occupational status), respondents with chronic diseases as well as those with private health insurance more often declared (*p* < 0.05) that they had visited a doctor during the COVID-19 pandemic (past 12 months). Detailed characteristics of the study population by visiting a doctor in the past 12 months is presented in Table 1.

**Table 1:** Characteristics of the study population by visiting a doctor in the past 12 months (*n* = 102,928).

	**Total sample**	**Visiting a doctor in the past 12 months**		
	***n*** **=102,928**		
		**Yes**	**No**	***p***
		***n*** **= 72,471**	***n*** **= 30,457**	
**Variable**	***n*** **(%)**	***n*** **(%)**	***n*** **(%)**	
**Gender**				
female	58,904 (57.2)	44,075 (74.8)	14,829 (25.2)	<0.001
male	44,024 (42.8)	28,396 (64.5)	15,628 (35.5)	
**Age (years)**				
18–29	8,868 (8.6)	5,309 (59.9)	3,559 (40.1)	<0.001
30–39	23,928 (23.2)	17,379 (72.6)	6,549 (27.4)	
40–49	25,159 (24.4)	17,968 (71.4)	7,191 (28.6)	
50–59	18,681 (18.1)	12,823 (68.6)	5,858 (31.4)	
60–69	19,013 (18.5)	13,519 (71.1)	5,494 (28.9)	
70+	7,279 (7.1)	5,473 (75.2)	1,806 (24.8)	
**Educational level**				
Primary	2,356 (2.3)	1,533 (65.1)	823 (34.9)	<0.001
Vocational	10,051 (9.8)	6,757 (67.2)	3,294 (32.8)	
Secondary	39,603 (38.5)	27,247 (68.8)	12,356 (31.2)	
Higher	50,918 (49.5)	36,934 (72.5)	13,984 (27.5)	
**Place of residence**				
Rural	21,353 (20.7)	16,508 (70.7)	6,825 (29.3)	0.052
City up to 50,000 residents	22,306 (21.7)	8,967 (70.2)	3,806 (29.8)	
City from 51,000 to 100,000 residents	12,807 (12.4)	7,318 (70.7)	3,038 (29.3)	
City from 101,000 to 200,000 residents	10,356 (10.1)	8,911 (69.6)	3,896 (30.4)	
City from 201,000 to 500,000 residents	12,773 (12.4)	15,615 (70.0)	6,691 (30.0)	
City above 500,000 residents	23,333 (22.7)	15,152 (71.0)	6,201 (29.0)	
**Occupational status**				
Active	77,429 (75.2)	53,633 (69.3)	23,796 (30.7)	<0.001
Passive	25,499 (24.8)	18,838 (73.9)	6,661 (26.1)	
**Presence of chronic diseases**				
Yes	43,608 (42.4)	35,286 (80.9)	8,322 (19.1)	<0.001
No	59,320 (57.6)	37,185 (62.7)	22,135 (37.3)	
**Private health insurance coverage**				
Yes	42,573 (41.4)	30,900 (72.6)	11,673 (27.4)	<0.001
No	60,355 (58.6)	41,571 (68.9)	18,784 (31.1)	

### Barriers to accessing health services during the COVID-19 pandemic in Poland

Almost half of adults in Poland (49.7%) declared that in the last 12 months, they had to visit a doctor, but this was not possible due to the barriers to accessing health services in Poland (Table 2). Out of 51,105 respondents who had experienced barriers to accessing health services during the COVID-19 pandemic, only 54.3% had visited a doctor in the past 12 months.

**Table 2:** Barriers to accessing health services during the COVID-19 pandemic by sociodemographic factors (*n* = 102,928).

**Variable**	**Barriers to accessing health services during the COVID-19 pandemic — the percentage of respondents who answered “Yes” by sociodemographic factors**									
	**Experience of barriers to accessing health services during the COVID-19 pandemic**		**The healthcare facility was closed due to the COVID-19 pandemic or transferred into a COVID-19 dedicated center**		**Too long waiting time**		**Financial barrier**		**Distance to the medical facility or problems with transport to the doctor**	
	***n*** **(%)**	***p***	***n*** **(%)**	***p***	***n*** **(%)**	***p***	***n*** **(%)**	***p***	***n*** **(%)**	***p***
**Overall**	51,105 (49.7)		29,622 (28.8)		40,629 (39.5)		24,420 (23.7)		8,378 (8.1)	
**Gender**										
Female	31,343 (53.2)	<0.001	17,350 (29.5)	<0.001	24,973 (42.4)	<0.001	16,443 (27.9)	<0.001	5,583 (9.5)	<0.001
Male	19,762 (44.9)		12,272 (27.9)		15,656 (35.6)		7,977 (18.1)		2,795 (6.3)	
**Age (years)**										
18–29	4,493 (50.7)	<0.001	2,382 (26.9)	<0.001	3,442 (38.8)	<0.001	2,358 (26.6)	<0.001	948 (10.7)	<0.001
30–39	12,545 (52.4)		7,364 (30.8)		10,072 (42.1)		5,817 (24.3)		2,165 (9.0)	
40–49	12,628 (50.2)		7,532 (29.9)		10,043 (39.9)		6,059 (24.1)		1,978 (7.9)	
50–59	8,971 (48.0)		5,303 (28.4)		7,081 (37.9)		4,392 (23.5)		1,378 (7.4)	
60–69	8,883 (46.7)		5,138 (27.0)		7,091 (37.3)		4,084 (21.5)		1,303 (6.9)	
70+	3,585 (49.3)		1,903 (26.1)		2,900 (39.8)		1,710 (23.5)		597 (8.2)	
**Educational level**										
Primary	1,220 (51.8)	0.09	674 (28.6)	<0.001	911 (38.7)	<0.001	689 (29.2)	<0.001	354 (15.0)	<0.001
Vocational	4,964 (49.4)		2,985 (29.7)		3,801 (37.8)		2,511 (25.0)		992 (9.9)	
Secondary	19,753 (49.9)		11,697 (29.5)		15,493 (39.1)		9,746 (24.6)		3,254 (8.2)	
Higher	25,168 (49.4)		14,266 (28.0)		20,424 (40.1)		11,474 (22.5)		3,778 (7.4)	
**Place of residence**										
Rural	10,497 (49.2)	<0.001	6,262 (29.3)	<0.001	8,044 (37.7)	<0.001	5,097 (23.9)	<0.001	2,418 (11.3)	<0.001
City up to 50,000 residents	10,745 (48.2)		6,436 (28.9)		8,424 (37.8)		4,892 (21.9)		1,940 (8.7)	
City from 51,000 to 100,000 residents	6,481 (50.6)		3,946 (30.8)		5,154 (40.2)		3,119 (24.4)		964 (7.5)	
City from 101,000 to 200,000 residents	5,277 (51.0)		3,093 (29.9)		4,208 (40.6)		2,554 (24.7)		696 (6.7)	
City from 201,000 to 500,000 residents	6,382 (50.0)		3,608 (28.2)		5,146 (40.3)		3,062 (24.0)		742 (5.8)	
City above 500,000 residents	11,723 (50.2)		6,277 (26.9)		9,653 (41.4)		5,696 (24.4)		1,618 (6.9)	
**Occupational status**										
Active	38,246 (49.4)	0.004	22,535 (29.1)	<0.001	30,571 (39.5)	0.9	17,770 (23.0)	<0.001	5,846 (7.6)	<0.001
Passive	12,859 (50.4)		7,087 (27.8)		10,058 (39.4)		6,650 (26.1)		2,532 (9.9)	
**Presence of chronic diseases**										
Yes	24,808 (56.9)	<0.001	14,123 (32.4)	<0.001	19,997 (45.9)	<0.001	12,636 (29.0)	<0.001	4,469 (10.2)	<0.001
No	26,297 (44.3)		15,499 (26.1)		20,632 (34.8)		11,784 (19.9)		3,909 (6.6)	
**Private health insurance coverage**										
Yes	20,950 (49.2)	0.02	12,399 (29.1)	0.04	16,896 (39.7)	0.2	9,013 (21.2)	<0.001	3,290 (7.7)	<0.001
No	30,155 (50.0)		17,223 (28.5)		23,733 (39.3)		15,407 (25.5)		5,088 (8.4)	
**Visiting a doctor in the past 12 months**										
Yes	39,324 (54.3)	<0.001	22,390 (30.9)	<0.001	31,791 (43.9)		18,710 (25.8)	<0.001	6,355 (8.8)	<0.001
No	11,781 (38.7)		7,232 (23.7)		8,838 (29.0)		5,710 (18.7)		2,023 (6.6)	

Out of all respondents (*n* = 102,928), 39.5% declared that they had to visit a doctor, but this was not possible due to too long waiting time (queues). Moreover, 28.8% of respondents indicated that they had to visit a doctor, but this was not possible because the healthcare facility was closed due to the COVID-19 pandemic or transferred into a COVID-19 dedicated medical facility (Table 2). Almost one-quarter of respondents (23.7%) declared that they had to visit a doctor, but this was not possible due to financial reasons (cost of medical services). Moreover, 8.1% of respondents indicated that they had to visit a doctor, but this was not possible due to distance to the medical facility or problems with transport to the doctor (Table 2). Out of four potential barriers to accessing health services that were analyzed in this study, only one barrier was mentioned by 17.5% of respondents, 17.7% experienced two different barriers, 10.6% of respondents experienced three different barriers to accessing health services and 3.8% of respondents indicated that they experienced all four barriers analyzed in this study.

Females, younger respondents, those living in big cities, currently unemployed individuals (passive occupational status), respondents with chronic diseases as well as those without private health insurance more often declared (*p* < 0.05) that they had experienced barriers to accessing health services during the COVID-19 pandemic (Table 2).

However, the type of barriers differed depending on socioeconomic factors (Table 2). Respondents aged 30–49 years more than other age groups declared that they had to visit a doctor, but this was not possible because the healthcare facility was closed due to the COVID-19 pandemic or transferred into a COVID-19 dedicated medical facility. Moreover, those who lived in rural areas or cities below 100,000 residents more often indicated medical facility closure or transformation into a COVID-19 dedicated center compared to those who lived in large cities (*p* < 0.05).

Too long waiting time was the major barrier to accessing health services which was indicated mostly by individuals with higher education as well as those who lived in the largest cities (>500,000 residents).

The financial barrier was mostly indicated by the youngest respondents (18–29 years), those with primary or vocational education, as well as currently unemployed individuals, and those who are not covered by private health insurance (Table 2).

Distance to the medical facility or problems with transport to the doctor was mostly indicated by the youngest respondents (18–29 years), individuals with primary education, those who lived in rural areas, and currently unemployed individuals (Table 2).

### Factors associated with accessing health services during the COVID-19 pandemic

The results of the multivariable logistic regression analyses are presented in Tables 3, 4. Females compared to males had higher odds of using health services in the past 12 months (OR: 1.52, 95% CI: 1.47–1.56; *p* = 0.01). Respondents aged 30 years and over had higher odds of using health services in the past 12 months compared to those aged 18–29 years (*p* < 0.05). Moreover, having higher education (OR: 1.31, 95% CI: 1.20–1.44; *p* < 0.001), living in rural areas (OR: 1.10, 95% CI: 1.06–1.15; *p* < 0.001), being unemployed (passive occupational status) (OR: 1.14, 95% CI: 1.09–1.18; *p* < 0.001), having chronic diseases (OR: 2.53, 95% CI: 2.45–2.61; *p* < 0.001) as well as having private health insurance (OR: 1.30, 95% CI: 1.26–1.33; *p* < 0.001) were significantly associated with higher odds of using health services in the past 12 months (Table 3).

**Table 3:** Factors associated with the use of health services in the past 12 months among adults in Poland (*n* = 102,928) - multivariable logistic regression model.

	**Factors associated with the use of health services in the past 12 months among adults in Poland**	
**Variable**	***p*** **-value**	**OR (95% CI)**
**Gender**		
Female	0.01	1.52 (1.47–1.56)
Male		Reference
**Age (years)**		
18–29		Reference
30–39	<0.001	1.71 (1.62–1.80)
40–49	<0.001	1.51 (1.42–1.59)
50–59	<0.001	1.18 (1.11–1.24)
60–69	<0.001	1.19 (1.13–1.26)
70+	<0.001	1.42 (1.42–1.32)
**Educational level**		
Primary		Reference
Vocational	0.046	1.11 (1.00–1.22)
Secondary	0.005	1.14 (1.04–1.25)
Higher	<0.001	1.31 (1.20–1.44)
**Place of residence**		
Rural	<0.001	1.10 (1.06–1.15)
City up to 50,000 residents	0.1	1.03 (0.99–1.08)
City from 51,000 to 100,000 residents	0.5	0.98 (0.94–1.03)
City from 101,000 to 200,000 residents	0.2	1.03 (0.98–1.09)
City from 201,000 to 500,000 residents	0.9	1.00 (0.95–1.05)
City above 500,000 residents		Reference
**Occupational status**		
Active		Reference
Passive	<0.001	1.14 (1.09–1.18)
**Presence of chronic diseases**		
Yes	<0.001	2.53 (2.45–2.61)
No		Reference
**Private health insurance coverage**		
Yes	<0.001	1.30 (1.26–1.33)
No		Reference

**Table 4:** Factors associated with the experience of barriers to accessing health services during the COVID-19 pandemic among adults in Poland (*n* = 102,928) - multivariable logistic regression model.

**Variable**	**Factors associated with the experience of barriers to accessing health services during the COVID-19 pandemic among adults in Poland**									
	**Experience of barriers to accessing healthcare services during the COVID-19 pandemic**		**Healthcare facility was closed due to the COVID-19 pandemic or transferred into COVID-19 dedicated medical facility**		**Too long waiting time**		**Financial barrier**		**Distance to the medical facility or problems with transport to the doctor**	
	***p*** **-value**	**OR** **(95% CI)**	***p*** **-value**	**OR** **(95% CI)**	***p*** **-value**	**OR** **(95% CI)**	***p*** **-value**	**OR** **(95% CI)**	***p*** **-value**	**OR** **(95% CI)**
**Gender**										
Female	<0.001	1.34 (1.30–1.37)	<0.001	1.06 (1.03–1.09)	<0.001	1.28 (1.25–1.32)	<0.001	1.66 (1.60–1.71)	<0.001	1.46 (1.39–1.54)
Male	Ref.	Ref.	Ref.	Ref.	Ref.	Ref.	Ref.	Ref.	Ref.	Ref.
**Age (years)**										
18–29	<0.001	1.32 (1.22–1.41)	<0.001	1.15 (1.06–1.24)	<0.001	1.14 (1.06–1.22)	<0.001	1.55 (1.43–1.68)	<0.001	1.77 (1.58–1.99)
30–39	<0.001	1.41 (1.32–1.49)	<0.001	1.38 (1.29–1.48)	<0.001	1.28 (1.20–1.36)	<0.001	1.43 (1.34–1.54)	<0.001	1.58 (1.42–1.76)
40–49	<0.001	1.22 (1.15–1.29)	<0.001	1.28 (1.20–1.37)	<0.001	1.11 (1.05–1.18)	<0.001	1.33 (1.24–1.43)	<0.001	1.29 (1.16–1.43)
50–59	0.5	1.02 (0.96–1.09)	<0.001	1.12 (1.05–1.20)	0.1	0.95 (0.90–1.02)	<0.001	1.16 (1.08–1.24)	0.2	1.08 (0.96–1.20)
60–69	0.02	0.92 (0.87–0.97)	0.2	1.04 (0.98–1.11)	<0.001	0.90 (0.85–0.96)	0.01	0.92 (0.86–0.98)	0.001	0.84 (0.76–0.94)
70+	Ref.	Ref.	Ref.	Ref.	Ref.	Ref.	Ref.	Ref.	Ref.	Ref.
**Educational level**										
Primary	0.02	1.10 (1.01–1.20)	0.2	1.06 (0.96–1.16)	0.5	0.97 (0.89–1.06)	<0.001	1.36 (1.24–1.50)	<0.001	1.91 (1.69–2.15)
Vocational	<0.001	1.09 (1.04–1.14)	<0.001	1.12 (1.07–1.18)	0.9	1.00 (0.95–1.04)	<0.001	1.26 (1.20–1.33)	<0.001	1.38 (1.28–1.49)
Secondary	<0.001	1.06 (1.04–1.09)	<0.001	1.11 (1.08–1.14)	0.6	1.01 (0.98–1.04)	<0.001	1.16 (1.13–1.20)	<0.001	1.10 (1.04–1.15)
Higher	Ref.	Ref.	Ref.	Ref.	Ref.	Ref.	Ref.	Ref.	Ref.	Ref.
**Place of residence**										
Rural	Ref.	Ref.	<0.001	1.12 (1.08–1.17)	Ref.	Ref.	Ref.	Ref.	<0.001	1.66 (1.55–1.77)
City up to 50,000 residents	0.9	1.00 (0.96–1.04)	<0.001	1.12 (1.07–1.16)	0.2	1.03 (0.99–1.07)	0.03	0.95 (0.91–0.99)	<0.001	1.39 (1.25–1.43)
City from 51,000 to 100,000 residents	0.002	1.07 (1.03–1.12)	<0.001	1.21 (1.16–1.27)	<0.001	1.12 (1.07–1.17)	0.04	1.06 (1.00–1.11)	0.03	1.10 (0.88–1.06)
City from 101,000 to 200,000 residents	<0.001	1.09 (1.04–1.14)	<0.001	1.16 (1.10–1.22)	<0.001	1.13 (1.08–1.19)	0.01	1.07 (1.01–1.13)	0.5	0.97 (0.88–1.06)
City from 201,000 to 500,000 residents	0.2	1.03 (0.99–1.08)	0.007	1.07 (1.02–1.12)	<0.001	1.11 (1.06–1.16)	0.4	1.03 (0.97–1.08)	<0.001	0.82 (0.75–0.89)
City above 500,000 residents	0.03	1.04 (1.01–1.08)	Ref.	Ref.	<0.001	1.15 (1.11–1.20)	0.005	1.07 (1.02–1.12)	Ref.	Ref.
**Occupational status**										
Active	0.9	1.00 (0.96–1.03)	0.008	1.05 (1.01–1.10)	0.01	1.05 (1.01–1.09)	Ref.	Ref.	Ref.	Ref.
Passive	Ref.	Ref.	Ref.	Ref.	Ref.	Ref.	<0.001	1.10 (1.06–1.14)	<0.001	1.33 (1.26–1.42)
**Presence of chronic diseases**										
Yes	<0.001	1.74 (1.69–1.78)	<0.001	1.43 (1.39–1.47)	<0.001	1.65 (1.61–1.69)	<0.001	1.66 (1.61–1.71)	<0.001	1.71 (1.63–1.80)
No	Ref.	Ref.	Ref.	Ref.	Ref.	Ref.	Ref.	Ref.	Ref.	Ref.
**Private health insurance coverage**										
Yes	0.4	1.00 (0.96–1.01)	0.1	1.02 (0.99–1.05)	0.07	1.02 (0.99–1.05)	<0.001	0.82 (0.79–0.84)	0.4	0.98 (0.94–1.03)
No	Ref.	Ref.	Ref.	Ref.	Ref.	Ref.	Ref.	Ref.	Ref.	Ref.

Ref. – reference category.

Females, individuals aged 18–49 years, those without higher education, respondents who lived in cities between 51,000 and 200,000 residents or the largest cities (above 500,000 residents), as well as those who had at least one chronic disease had significantly (*p* < 0.05) higher odds of experiencing barriers to accessing health services during the COVID-19 pandemic (Table 4). Out of four different barriers to accessing health services analyzed in this study, females had higher odds of experiencing these barriers when compared to males (Table 4). Moreover, individuals aged 18–49 had higher odds of experiencing all four barriers when compared to older groups (*p* < 0.001). Respondents without higher education had higher odds of experiencing financial barriers or transportation barriers compared to those with higher education (*p* < 0.001). Those who lived in rural areas or small cities with up to 50,000 residents had higher odds of experiencing transportation barriers or barriers resulting from the closure of medical facilities due to the COVID-19 pandemic, compared to those who lived in large cities above 500,000 residents (Table 4). Detailed characteristics of factors associated with each of the analyzed barriers to accessing health services is presented in Table 4.

## Discussion

This is the first study on public perception of barriers to accessing health services during the COVID-19 pandemic, that was carried out on a large sample of more than 100 thousand adults in Poland. This study was carried out between October and December 2021, during the fourth wave of the COVID-19 pandemic in Poland. Findings from this study showed that almost half of adults in Poland reported barriers to accessing health services during the COVID-19 pandemic. More than 45% of adults in Poland resigned from visiting a doctor despite health needs due to the occurrence of barriers to accessing health services. More than one-quarter of adults in Poland indicated that they had to visit a doctor, but this was not possible because the healthcare facility was closed due to the COVID-19 pandemic or transferred into a COVID-19 dedicated medical facility. Findings from this study suggest that increasing the hospital capacity of COVID-19 patients in Poland had a negative impact on the status of patients with diseases other than COVID-19.

Access to healthcare is a basic human need (28). National health systems should provide access to health services. In Poland, all the individuals covered with mandatory health insurance should have free access to healthcare. All individuals are obligated to register in primary care practice (choose their general practitioner) (20). Moreover, in case of emergency, all insured has access to specialist hospital-based or ambulatory care (20, 21).

As parents are obligated to take care of their children, in this study respondents were asked about visiting a healthcare facility because of their health problem or their child's illness or health condition. Findings from this study showed that during the COVID-19 pandemic, 70.5% of adults in Poland had visited a doctor. Previously published data showed that females have higher medical care service utilization than males (29). Findings from this study confirmed that even during the COVID-19 pandemic, females were more likely to visit a doctor. The highest healthcare service utilization was observed among those aged 30–49 years, as well as those over 70 years. We can hypothesize that a markable proportion of Poles aged 30–49 years, had visited a doctor due to the child's illness or health condition. Age is a significant factor affecting the risk of diseases, so older people are more likely to visit a doctor (30). Moreover, healthcare utilization increased with the level of education. Educational level is associated with the health literacy level as well as personal health behaviors (31). Individuals who lived in rural areas were more likely to visit a doctor when compared to those who lived in cities. We can hypothesize that local rural communities have better relations with their doctors or shorter waiting time, so they may be more likely to visit a doctor. As expected, respondents with chronic diseases were more likely to visit a doctor in the past 12 months. The presence of chronic disease is a significant factor associated with higher healthcare utilization (32). In this study, individuals with private (complementary) health insurance were more likely to visit a doctor. Private health insurance is gaining popularity, mostly due to the shorter waiting times as well as the quality of care (21).

According to the WHO estimates, partial or complete suspension of regular health services in the field of hypertension, diabetes, and diabetes-related complications, cancer, and cardiovascular outbreaks due to the COVID-19 pandemic were observed in 53% of countries worldwide (13, 33). Previously published data indicated a markable decrease in urological and diabetes care utilization in Poland (23–25). Findings from this study showed that half of the adults in Poland declared that they had to visit a doctor, but this was not possible due to barriers to accessing health services during the COVID-19 pandemic. Females, individuals aged 18–49 years, those without higher education, inhabitants of cities 51,000 to 200,000 residents or cities above 500,000 residents as well as those with chronic diseases were more likely to report barriers to access healthcare during the pandemic. We can hypothesize that these groups are at higher risk of health inequalities caused by the pandemic. Moreover, these groups should be considered as a target population for public health programs aimed to reduce health debt caused by COVID-19.

During the pandemic, numerous hospitals in Poland were transformed into COVID-19 dedicated centers (8). Moreover, a markable proportion of general practitioners preferred teleconsultations as a type of medical care offered to patients. Findings from this study showed, that more than one-quarter of respondents indicated that the closure of the medical facility or transformation into a COVID-19 dedicated center was a barrier to accessing health services during the pandemic. Limited healthcare resources (including human resources) may worsen access to healthcare. Healthcare resources management requires balanced actions that do not seek to restrict access to one group of patients in favor of patients with other diseases. In this study, individuals under 60 years of age were more likely to report the closure of the medical facility or transformation into a COVID-19 dedicated center as a barrier to accessing health services. Moreover, this barrier was also indicated by those who lived in rural areas or cities below 500,000 residents. In large cities, there are numerous healthcare facilities, so those who lived in large cities may visit other healthcare facilities when their preferred place is closed. In rural areas, there are single centers so the closure of these centers may deprive many people of access to healthcare (34).

Long waiting time for publicly funded health services (especially long patient queues for specialist doctors) is one of the most common problems indicated by the public (35). To reduce the within time, numerous Poles use private health services (21). The COVID-19 pandemic has affected both public and private healthcare. Waiting time also depends on the medical condition that is the purpose of the medical visit. In this study, individuals aged 18–49 years, as well as those aged 60–69 years, were more likely to indicate the long waiting time as a barrier to accessing health services. We can hypothesize that the type of health problems presented in these groups (e.g., respiratory infections among children of parents aged 18–49 years or non-communicable chronic diseases among those aged 60–69 years) are very common, so the waiting time for the medical consultation is long. Moreover, occupationally active individuals were more likely to indicate the long waiting time as a barrier. We can hypothesize that this group is busy due to occupational duties, so fast access to healthcare is important to maintain productivity. This hypothesis is supported by the fact, that private medical insurance is mostly offered to occupationally active individuals (e.g., as a part of the motivational package). Moreover, waiting time was indicated by those who lived in cities above 500,000 residents. We can hypothesize that in the largest cities with medical universities, patients are more likely to visit clinics and medical practices of physicians with a scientific background, so the waiting time for these highly-qualifies individuals is longer.

Females, individuals aged 18–49 years, those without higher education, occupationally passive individuals, as well as those with chronic diseases were more likely to report financial barriers or problems with transport to the doctor. Moreover, place of residence also affected the access to health services. Socioeconomic status is a significant determinant of health that may also affect health inequalities (36, 37). Financial barriers, as well as transportation, are one of the common barriers to accessing health services that were also reported before the COVID-19 pandemic. Public health authorities as well as policymakers should reduce these barriers, which may be easily removed by the organization of healthcare that will consider socioeconomic determinants of health (37).

This study has numerous practical implications for public health in Poland and may be used as a benchmark for other Europe countries. First, this study confirmed that Poles had limited access to health care during the COVID-19 pandemic and estimated the magnitude of the problem. Due to the barriers to accessing health services, there was a phenomenon of the so-called health debt. The long-term consequences of limited access to health services during the COVID-19 pandemic are not yet known. This can worsen the health status of the population and delay or overlook the diagnosis of diseases, which has negative consequences at both the individual and population levels. Second, the results showed significant differences in the public perception of barriers to accessing health services during the COVID-19 pandemic by gender, age, place of residence, occupational activity, or health status. Efforts should be made to reduce inequalities in access to health services, especially by place of residence. Third, groups that were most affected by the limited access to health services presented in this study may be used by public health authorities to prepare health policies that will aim to reduce health inequalities deteriorated by the COVID-19 pandemic.

There are several limitations of this study. Barriers to accessing health services were identified through self-reported data. Medical records were not verified, as we used cross-sectional survey methods. The list of potential barriers to accessing health services was limited to the four most common barriers that were selected by the medical experts. Further research (especially qualitative studies) is needed to better understand this problem. Data were collected with computer-assisted web interviews, so the study sample is limited to Internet users. Nevertheless, more than 90% of households in Poland have an Internet connection (38). The question about the purpose of the visit to medical facilities was not addressed so analysis by medical conditions is unavailable.

## Conclusions

This study confirmed that the COVID-19 pandemic has worsened access to health services in Poland. During the pandemic, new barriers to accessing health services, such as the temporary closure of healthcare facilities for non-COVID patients were revealed. A markable proportion of adults in Poland resigned from visiting a doctor despite health needs due to the occurrence of barriers to accessing health services, which may significantly deteriorate the health status of the population. In addition, the inequalities in access to health services due to sociodemographic factors observed in this study may widen due to barriers to accessing health services during the COVID-19 pandemic. Multidisciplinary strategies should be developed to ensure universal access to health services for different social groups in Poland.

## Data availability statement

The raw data supporting the conclusions of this article will be made available by the authors, without undue reservation.

## Ethics statement

The studies involving human participants were reviewed and approved by the Ethical Review Board at the Medical University of Warsaw, Warsaw, Poland (no. AKBE/149/2021). The patients/participants provided their written informed consent to participate in this study.

## Author contributions

All authors PM-T, AZ, MG, MJ, IB, AW, and JK have contributed significantly to this work, have seen the contents of the manuscript, and agreed to its submission.

## Funding

Publication was funded by Medical University of Lodz, Department of Social Medicine (project no. 503/6-029-01/503-61-001-19-00).

## Conflict of interest

The authors declare that the research was conducted in the absence of any commercial or financial relationships that could be construed as a potential conflict of interest.

## Publisher's note

All claims expressed in this article are solely those of the authors and do not necessarily represent those of their affiliated organizations, or those of the publisher, the editors and the reviewers. Any product that may be evaluated in this article, or claim that may be made by its manufacturer, is not guaranteed or endorsed by the publisher.
